# Characterization of a Long Non-Coding RNA, the Antisense RNA of Na/K-ATPase α1 in Human Kidney Cells

**DOI:** 10.3390/ijms19072123

**Published:** 2018-07-21

**Authors:** Xiaoming Fan, Usman M. Ashraf, Christopher A. Drummond, Huilin Shi, Xiaolu Zhang, Sivarajan Kumarasamy, Jiang Tian

**Affiliations:** 1 Department of Medicine, University of Toledo, Toledo, OH 43614, USA; xiaoming.fan@rockets.utoledo.edu (X.F.); cdrummo88@gmail.com (C.A.D.); Huilin.Shi@UToledo.Edu (H.S.); Xiaolu.Zhang@utoledo.edu (X.Z.); 2 Department of Physiology and Pharmacology, Center for Hypertension and Personalized Medicine, University of Toledo, Toledo, OH 43614, USA; Usman.Ashraf2@rockets.utoledo.edu (U.M.A.); Sivarajan.Kumarasamy@utoledo.edu (S.K.); 3 MPI Research, Mattawan, MI 49071, USA

**Keywords:** long non-coding RNA, ATP1A1-AS1, Na/K-ATPase, Src, signaling transduction

## Abstract

Non-coding RNAs are important regulators of protein-coding genes. The current study characterized an antisense long non-coding RNA, *ATP1A1-AS1*, which is located on the opposite strand of the Na/K-ATPase α1 gene. Our results show that four splice variants are expressed in human adult kidney cells (HK2 cells) and embryonic kidney cells (HEK293 cells). These variants can be detected in both cytosol and nuclear fractions. We also found that the inhibition of DNA methylation has a differential effect on the expression of *ATP1A1-AS1* and its sense gene. To investigate the physiological role of this antisense gene, we overexpressed the *ATP1A1-AS1* transcripts, and examined their effect on Na/K-ATPase expression and related signaling function in human kidney cells. The results showed that overexpression of the *ATP1A1-AS1-203* transcript in HK2 cells reduced the Na/K-ATPase α1 (*ATP1A1*) gene expression by approximately 20% (*p* < 0.05), while reducing the Na/K-ATPase α1 protein synthesis by approximately 22% (*p* < 0.05). Importantly, overexpression of the antisense RNA transcript attenuated ouabain-induced Src activation in HK2 cells. It also inhibited the cell proliferation and potentiated ouabain-induced cell death. These results demonstrate that the *ATP1A1-AS1* gene is a moderate negative regulator of Na/K-ATPase α1, and can modulate Na/K-ATPase-related signaling pathways in human kidney cells.

## 1. Introduction

Recent advances in genome-wide sequencing has led to the transcription of the majority of the human genome, revealing that only a small portion of the transcribed RNAs actually codes proteins [1,2,3]. The RNAs lacking protein coding information were defined as non-coding RNAs, which, depending on their length, were further divided into short non-coding RNAs (less than 200 nucleotides) and long non-coding RNAs (lncRNAs) [4,5,6]. lncRNAs have structures of introns and exons like most messenger RNAs (mRNAs). lncRNAs could also have polyA tail and splice variants [3,7]. However, the expression level of lncRNAs is much lower comparing to that of mRNA, rRNA, or tRNA. It was estimated that lncRNAs only make up 0.03–0.2% of total RNA by mass [8]. Many of the discovered lncRNAs are located on the antisense strand of well-defined transcriptional units, which are usually called natural antisense RNAs [9]. Some identified natural antisense genes were found to regulate their sense protein-coding gene expression [10] or to act in cellular process such as regulating cell signaling or cell-cell communication [11,12]. However, the physiological function of most lncRNAs or natural antisense RNAs have not been elucidated.

The *ATP1A1* antisense RNA (*ATP1A1-AS1*) is a natural antisense gene of Na/K-ATPase α1, *ATP1A1*. The transcription of this antisense gene was first reported in a human genomic study [13], which expresses at least four splice variants *(ATP1A1-AS1-201*, *ATP1A1-AS1-202*, *ATP1A1-AS1-203*, and *ATP1A1-AS1-204*). The sequence of *ATP1A1-AS1-203* and *ATP1A1-AS1-204* transcripts are partially complementary to the mRNA of sense *ATP1A1* gene. At least 27 human tissues, including kidney, heart, and blood, were demonstrated to express the *ATP1A1-AS1* RNA transcripts [14,15].

Na/K-ATPase is a transmembrane protein that was discovered in 1957, by Skou [16]. In addition to its canonical ion transporting function, Na/K-ATPase α1 was found to be associated with other signaling proteins and functions as a signal transducer [17,18,19]. Data from clinical research showed that cardiac contractility is positively correlated with Na/K-ATPase levels in heart failure patients [20,21]. It was also demonstrated that signaling mediated by Na/K-ATPase α1 regulates renal and cardiac cell survival *in vitro* and associates with renal and cardiac function in vivo [22,23,24,25,26,27]. Na/K-ATPase expression can be regulated by multiple transcriptional factors and a variety of chemical compounds [28]. The current work is aimed to characterize this *ATP1A1-AS1* gene, and its role in regulating the Na/K-ATPase α1 expression and its signaling function in human kidney cells.

## 2. Results

### 2.1. Differential Expression and Subcellular Distribution of ATP1A1-AS1 Splice Variants in Human Kidney Cells

**Figure 1:**
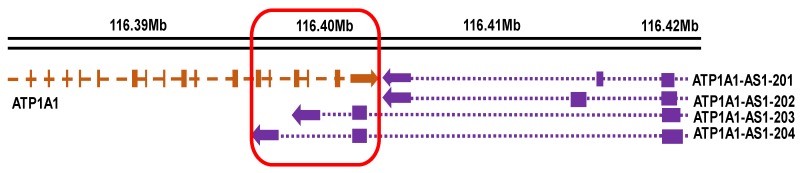
Schematic presentation of *ATP1A1* and *ATP1A1-AS1* gene. Sequence information was obtained from Ensembl (GRCh38). The vertical brown square indicates exons, and horizontal brown line indicates introns of the *ATP1A1* gene. Purple square indicates exons, and purple dots indicates introns of *ATP1A1-AS1* gene. The arrows indicate the transcriptional direction. The red square indicates the approximate overlapping sequences between *ATP1A1* and *ATP1A1-AS1*.

**Figure 2:**
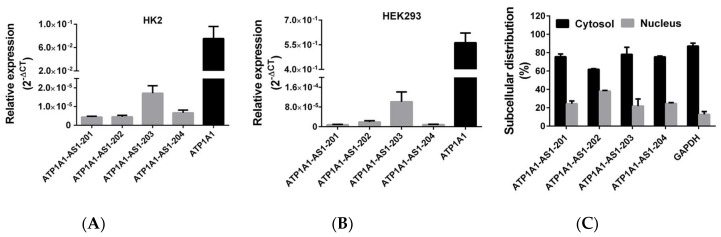
Differential expression and subcellular distribution of *ATP1A1-AS1* splice variants in human kidney cells. (**A**) Expression of four splice variants of ATP1A1-AS1 and messenger RNA (mRNA) level of *ATP1A1* in human adult kidney cells (HK2 cells). (**B**) Expression of *ATP1A1-AS1* splice variants and *ATP1A1* in human embryonic kidney cells (HEK293 cells). (**C**) Distribution of *ATP1A1-AS1* splice variants in cytosol and nuclear fraction of HK2 cells. *GAPDH* mRNA was used as cytoplasmic control RNA. These experiments were repeated four times.

The *ATP1A1-AS1* gene is located in the region of 116,392,247–116,418,622 on the reverse strand of human chromosome 1 (Figure 1) based on Ensembl GRCh38.p12 (http://www.ensembl.org). To assess the expression level of each splice variants, we synthesized specific primers corresponding to the four transcripts of *ATP1A1-AS1* as described in Material and Method section. As shown in Figure 2A, all 4 splice variants can be detected in human adult kidney cells (HK2 cell line), while the ATP1A1-AS1-203 expression is relatively higher than the other three transcripts. A similar expression pattern was observed in HEK293 cells, a human embryonic kidney cell line (Figure 2B). In both cell lines, the expression level of the antisense transcripts was approximately 5000 times lower than that of *ATP1A1*. We also examined the subcellular distribution of these antisense transcripts in HK2 cells. A commercial isolation kit was used to extract RNA from the cytosol and nuclear fraction from these cells. The mRNA of Glyceraldehyde 3-phosphate dehydrogenase (*GAPDH*) was used as a marker of cytosol RNA. As shown in Figure 2C, about 90% *GAPDH* mRNA was detected in the isolated cytosol fraction—suggesting that the separation of cytosol and nuclear was successful. The expression of *ATP1A1-AS1* can be detected in both cytosol and nuclear fraction, but the ratio was much higher in the cytosol fraction.

### 2.2. Epigenetic Regulation of ATP1A1-AS1 Expression

**Figure 3:**
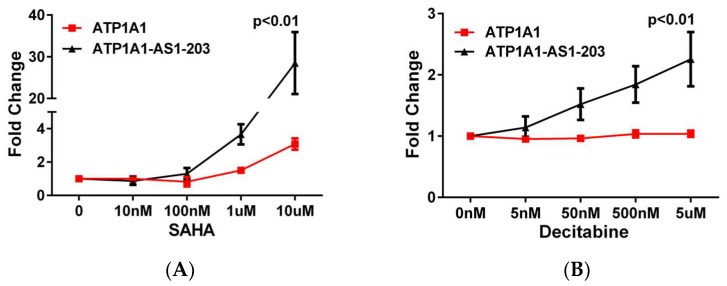
Epigenetic regulation of *ATP1A1-AS1* expression in HK2 cells. Cultured HK2 cells were treated with deacetylase inhibitor, suberoylanilide hydroxamic acid (SAHA) (**A**) or DNA methylation inhibitor Decitabine (**B**) for 48 h and the total RNA was extracted using a commercial RNA extraction kit as described in Method section. The expression of *ATP1A1-AS1-203* and *ATP1A1* was measured using RT-qPCR. These experiments were repeated four times. Data was analyzed using Two-way ANOVA.

To understand the regulation mechanism of *ATP1A1-AS1* expression, we treated HK2 cells with a histone deacetylase (HDAC) inhibitor, suberoylanilide hydroxamic acid (SAHA), at concentrations of 10 nM, 100 nM, 1 μM, and 10 μM for 48 h, and measured the *ATP1A1-AS1-203* levels as the representative of *ATP1A1-AS1* expression. As shown in Figure 3A, SAHA treatment at lower concentrations (10 and 100 nM) had no significant effect on the expression of the sense or antisense gene expression. However, SAHA at higher concentrations (1 and 10 μM) significantly increased the expression of *ATP1A1-AS1-203*. It also increased the expression of *ATP1A1*, but to a lesser degree. We also treated the cells with a DNA methylation inhibitor, Decitabine, at concentrations of 5 nM, 50 nM, 500 nM, and 5 μM for 48 h to test if DNA methylation regulates the *ATP1A1-AS1* expression. The result showed that inhibition of DNA methylation had significant effect on upregulating the antisense gene (*ATP1A1-AS1*) expression, but it did not affect the *ATP1A1* gene expression (Figure 3B). These results suggest a differential effect of methylation and acetylation on the regulation of the sense and antisense gene.

**Figure 4:**
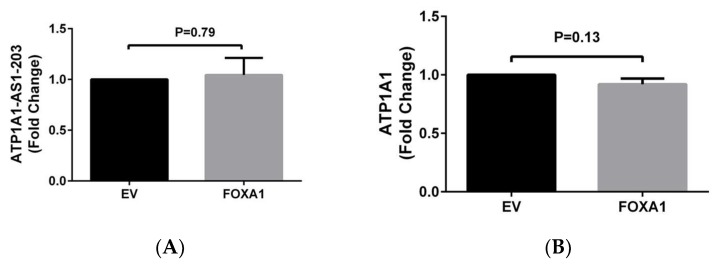
The effect of *FOXA1* overexpression on *ATP1A1-AS1* expression in HK2 cells. A pcDNA3.1(-B) plasmid that overexpresses *FOXA1* (FOXA1) or empty vector (EV) was transiently transfected into HK2 cells for 48 h, an empty pcDNA3.1(-B) plasmid was used as control. The expression of *ATP1A1-AS1-203* (**A**) and *ATP1A1* (**B**) were measured using RT-qPCR. These experiments were repeated six times. Data was analyzed using *t*-test.

In addition, we also identified a FOXA1 binding site on the upstream of *ATP1A1-AS1* gene based on the gene sequence information. To test if FOXA1 regulates the antisense gene expression, we cloned *FOXA1* cDNA sequence into a pcDNA3.1(-B) vector and transiently transfected into HK2 cells for 48 h. As shown in Figure 4, overexpression of *FOXA1* induced only a slight increase in the expression of *ATP1A1-AS1-203* and a slight decrease in the expression of *ATP1A1* in these cells, which had no statistical significance.

### 2.3. ATP1A1-AS1 Regulates the Sense Na/K-ATPase α1 Gene Expression and Protein Synthesis in HK2 Cells

**Figure 5:**
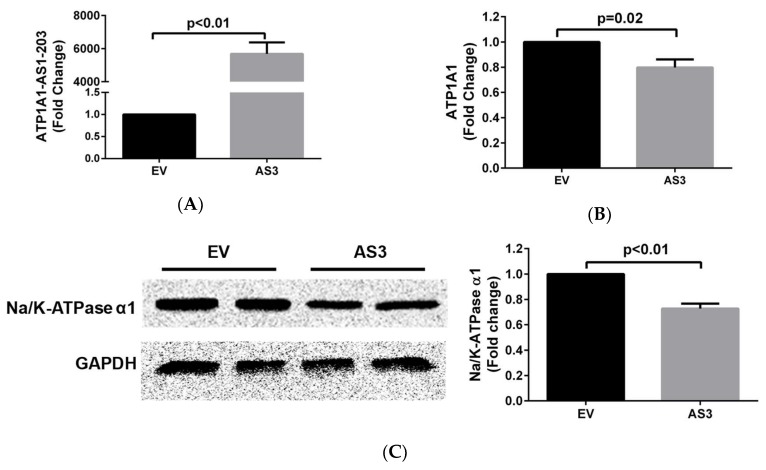
Overexpression of *ATP1A1-AS1-203* negatively regulates *ATP1A1* gene expression and Na/K-ATPase α1 protein level in HK2 cells. HK2 cells were transiently transfected with pcDNA3.1(-B) plasmid that overexpresses *ATP1A1-AS1-203* (AS3) or empty vector (EV) for 48 h and expression of *ATP1A1-AS1-203* (**A**) and *ATP1A1* (**B**) were measured using RT-qPCR. The Na/K-ATPase α1 protein level (**C**) was measured by Western blot using the Radioimmunoprecipitation assay (RIPA) buffer resolved cell lysates. These experiments were repeated 4 times. Data was analyzed using *t*-test.

To test the role of *ATP1A1-AS1* on the regulation of its sense gene, we constructed a plasmid vector that overexpresses *ATP1A1-AS1-203*, the highest RNA transcript of the *ATP1A1-AS1* gene, and transiently transfected into the cultured HK2 cells for 48 h. We measured the Na/K-ATPase α1 mRNA and protein levels using RT-qPCR and Western blot, respectively. As shown in Figure 5, overexpression of *ATP1A1-AS1-203* caused approximately 20% decrease (*p* < 0.05) in *ATP1A1* mRNA levels and 22% decrease in Na/K-ATPase α1 protein levels in these cells, indicating that the antisense gene is a negative regulator of Na/K-ATPase α1.

**Figure 6:**
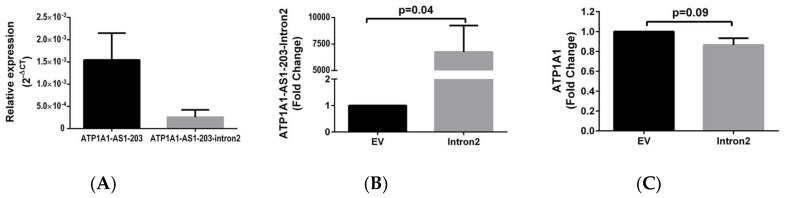
Effect of intron 2 of *ATP1A1-AS1-203* on the expression of *ATP1A1* in HK2 cells. (**A**) The expression level of *ATP1A1-AS1-203* and *ATP1A1-AS1-203*-Intron 2 in HK2 cells; (**B**,**C**) The effect of *ATP1A1-AS1-203*-Intron 2 overexpression on *ATP1A1* gene expression. The *ATP1A1-AS1-203*-Intron 2 overexpression vector (Intron2) was transiently transfected into HK2 cells for 48 h and the total RNA extracted from the cell lysates were used for RT-qPCR measurement of *ATP1A1-AS1-203*-Intron 2 (**B**) and *ATP1A1* (**C**). These experiments were repeated four times. Data was analyzed using *t*-test.

Since studies have indicated that introns of lncRNAs are also expressed and may be functionally important in a cell [29], we then examined the presence of the introns of *ATP1A1-AS1* transcript. Using RT-qPCR, we measured both *ATP1A1-AS1-203* and the intron 2 (between exon 2 and exon 3 of *ATP1A1-AS1-203*) expression level. As shown in Figure 6A, the intron 2 was detectable but its expression level was approximately six times lower than that of the *ATP1A1-AS1-203*. To test if intron 2 regulates Na/K-ATPase expression, HK2 cells were transiently transfected with pcDNA3.1(-B) vector containing sequence of intron 2 and compared with cells that transfected with empty vectors. The result showed that transient transfection of intron 2 vector for 48 h increased the intron 2 level by about 7000 fold (Figure 6B), whereas the expression of *ATP1A1* gene was decreased by about 14% (Figure 6C).

### 2.4. Overexpression of ATP1A1-AS1 Regulates Na/K-ATPase-Related Signaling and Cell Proliferation

**Figure 7:**
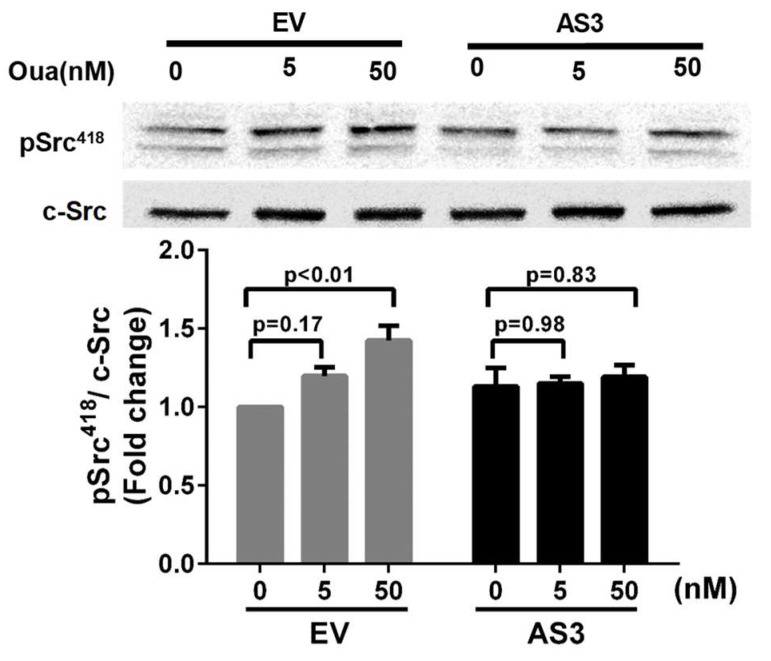
Overexpression of *ATP1A1-AS1-203* inhibits ouabain-induced Src activation in HK2 cells. HK2 cells were transfected with *ATP1A1-AS1-203* overexpressing vector (AS3) or empty vector (EV) for 24 h followed by ouabain (Oua) treatment for 15 min. Cell lysates were collected in RIPA buffer. The phospho-Src at Tyr418 (pSrc418) and c-Src were probed using Western blot. The upper panel was a representative Western blot, and the lower panel was the quantification result. These experiments were repeated four times. Data was analyzed using Two-way ANOVA.

Our previous studies, References [25,30,31], have shown that reduction of Na/K ATPase α1 attenuates cardiotonic steroids (CTS)-induced Src activation and potentiates CTS-induced cell growth inhibition. To examine the effect of *ATP1A1-AS1* on the Na/K-ATPase-related signaling function, we transiently transfected HK2 cells with the *ATP1A1-AS1-203* overexpression vector for 24 h, followed by ouabain treatment for 15 min. Empty vector transfected cells were used as control. The cell lysate was then collected to probe for Src phosphorylation at tyrosine 418 (pSrc^418^), an indicator of Src activation. As shown in Figure 7, ouabain treatment at 50 nM induced significant increase in Src phosphorylation in cells transfected with empty vector, whereas in cells that overexpress *ATP1A1-AS1-203*, ouabain failed to induce the Src activation.

**Figure 8:**
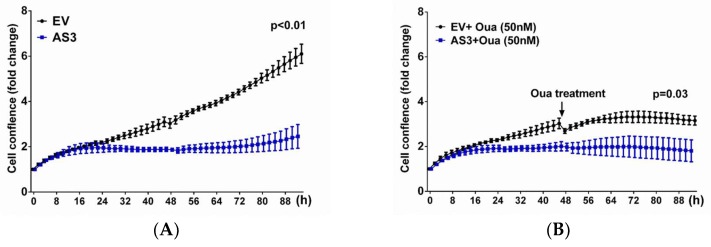
Overexpression of *ATP1A1-AS1-203* inhibits HK2 cell growth in presence or absence of ouabain. HK2 cells were transfected with *ATP1A1-AS1-203* vector (AS3) or empty vector (EV) for 48 h, followed by treatment of 50 nM ouabain (Oua) for another 48 h. Cells without ouabain treatment was used as control. Cell proliferation was monitored using an Incucyte incubator equipped with a camera that automatically taking pictures every 2 h at 9 different regions of each cell culture well. (**A**) Cell growth curve in AS3 or EV transfected HK2 cells without ouabain treatment; (**B**) Cell growth curve in AS3 or EV transfected HK2 cells that treated with 50 nM ouabain. These experiments were repeated four times. Data was analyzed using Two-way ANOVA.

To examine the role of *ATP1A1-AS1* on cell proliferation, we transfected the cells with *ATP1A1-AS1-203* overexpression vector. The cells were then incubated in an Incucyte incubator equipped with a camera to monitor the cell growth for 96 h. To investigate the cell growth in the presence of ouabain, HK2 cells transfected with *ATP1A1-AS1-203* overexpression vector for 48 h were treated with 50 nM ouabain for additional 48 h. Cell growth was also monitored for a total of 96 h. As shown in Figure 8, overexpression of *ATP1A1-AS1-203* inhibited cell proliferation in the absence or presence of ouabain. We also observed that addition of ouabain induced cell death in the *ATP1A1-AS1-203* overexpressed cells. These results are in line with our previous observations in the setting of decreased Na/K-ATPase α1 expression [24,25].

## 3. Discussion

In this report, we examined the splice variants and subcellular distribution of a newly identified antisense lncRNA, *ATP1A1-AS1*. Our result, for the first time, showed that *ATP1A1-AS1* is a potential negative regulator of its sense gene, Na/K-ATPase α1, in human kidney cells. Na/K-ATPase is a major component for sodium reabsorption in kidney, and its signaling functions are associated with renal function and kidney disease [27]. We have previously reported that reduction of Na/K-ATPase could significantly attenuate the Na/K-ATPase-related signaling in kidney proximal tubule cells, and potentiate the cell apoptosis [25,31]. A recent publication also demonstrated that impairment of Na/K-ATPase signaling may contribute to hyperuricemia-induced renal tubular injury [26]. Decrease of Na/K-ATPase is also a common phenomenon in patients with congestive heart failure [20,32], aging [33,34,35], diabetes with hypertension [36,37,38], and neurological disorders [39,40]. Consistently, the current work showed that increased expression of *ATP1A1-AS1* gene attenuated the ouabain-induced Src activation and inhibited cell growth or potentiate ouabain-induced cell death.

However, we noted that the regulatory effect of *ATP1A1-AS1* on the Na/K-ATPase α1 expression was moderate in human kidney cells. Overexpression of *ATP1A1-AS1* by a few thousand times in HK2 cells only caused about 20% change in Na/K-ATPase protein content. Whereas, it significantly attenuated ouabain-induced Src activation and inhibited cell growth. Our previous study showed that reduction of Na/K-ATPase α1 by 50–60% through siRNA transfection only partially blocked ouabain-induced Src activation in pig kidney proximal tubule cells [25,31]. These results suggest that other effects in addition to the Na/K-ATPase reduction may exist and contribute to the Src kinase regulation and cell proliferation when *ATP1A1-AS1* was overexpressed. It is also unclear whether Na/K-ATPase α1 is the only target of *ATP1A1-AS1*. Molecular mechanisms for antisense RNA-induced sense gene regulation are not fully understood. Mechanisms, such as RNA interference (RNAi) by formation of double strand RNA or steric clashes induced by antisense RNA, may exist to regulate the corresponding sense gene [41,42,43]. More recently, investigators hypothesized that the antisense RNA could control the quality and quantity of the sense gene by producing endogenous siRNAs [44]. In addition, our data showed that the alternative splicing of antisense gene exist in human kidney cells, which may also play a role in regulating the sense gene expression. Identifying these mechanisms in the future will provide more effective tools to manipulate the Na/K-ATPase α1 expression, and protect normal renal and cardiac function in humans.

Previous studies have shown that DNA methylation and histone acetylation modifications are important regulators of lncRNAs expression. In the current study, we observed that histone acetylation and DNA methylation had differential effects on *ATP1A1-AS1* and *ATP1A1* expression. The *ATP1A1-AS1* gene expression was more responsive to the epigenetic modification, especially to the change of DNA methylation, whereas the effect of DNA methylation on *ATP1A1* gene expression was modest. This observation is consistent with previous findings that DNA methylation change did not affect the *ATP1A1* expression [45,46]. However, based on the data released from ENCODE/HAIB study (https://www.ncbi.nlm.nih.gov/geo/info/ENCODE.html), in human kidney cells, there are 12 DNA methylation sites with seven methylated, one partially methylated, and four unmethylated on the *ATP1A1* coding strand. Whereas on the *ATP1A1-AS1* coding strand, there are ten DNA methylation sites with five methylated, one partially methylated and four unmethylated. Therefore, the number of methylation sites or methylated nucleotide number alone may not explain the differential regulation effect on this pair of sense/antisense gene. In addition, even though the sequence of *ATP1A1-AS1* data indicate a transcription factor binding site for FOXA1, our experimental data showed that overexpression of *FOXA1* failed to significantly regulate the *ATP1A1-AS1* expression.

In summary, the current findings showed that the *ATP1A1-AS1* can negatively regulate its sense gene expression, and affect the Na/K-ATPase signaling function in human kidney cells. However, it merits further studies to fully understand the physiological role of this antisense RNA.

## 4. Materials and Methods

### 4.1. Cell Culture

Human kidney cells (HK2 cell line and HEK293 cell line) were purchased from American Type Culture Collection (ATCC) and cultured in Dulbecco’s modified Eagle’s medium (HK2 cells) or Eagle’s minimum essential medium (HEK293 cells) supplemented with 10% fetal bovine serum (FBS), 100 units/mL penicillin, and 100 μg/mL streptomycin in a humidified incubator with 5% CO_2_.

### 4.2. Quantitation of ATP1A1-AS1 Expression Using RT-qPCR

Total RNA was extracted from cultured HK2 or HEK293 cells using RNeasy Mini Kit from Qiagen (Valencia, CA, USA) (Cat. No. 74104) following the protocol provided by the manufacturer. About 1 μg of extracted RNA was used for cDNA synthesis with the RT^2^ First Strand cDNA synthesis kit from Qiagen (Cat. No. 330404). Expression of *ATP1A1-AS1* were quantified by RT-qPCR. The primers used for RT-qPCR and the expected product size were as following: *ATP1A1-AS1-201* (forward: TTTGCGCTAACGATGAGAAC; reverse: GCATTTCCAGATGCATGGT, expected product size: 68 bp), *ATP1A1-AS1-202* (forward: GGGCTGAGAGCTAAGGAGTG; reverse: ATGGGCATTTCCTCCTGAT, expected product size: 134 bp), *ATP1A1-AS1-203* (forward: AGCGGTCATCCCAGTCCAC; reverse: CCAGTGTGTGTCCCAATCCC, expected product size: 136 bp), and *ATP1A1-AS1-204* (forward: GTTCTCAGCCAGAATCACAAACTT; reverse: GATGAGAGAAAGATACGCCAAAAT, expected product size: 130 bp), Intron 2 of *ATP1A1-AS1-203* (forward: GTCTCTGAAATCAACCTCAACC, reverse: ACTAAATTCCTTCTCCCCACC, expected product size: 242 bp). *GAPDH* was used as internal control. Primer pair for *GAPDH* was from Qiagen (Cat No. PPH00150F, expected product size: 130 bp). The expression level of each RNA transcript was presented as 2^−Δ*C*t^ (Δ*C*t is the difference of *C*t value between the specific RNA and mRNA of *GAPDH*). The fold change of the specific RNA transcript after treatment was calculated using the formula: Fold change = 2^−ΔΔ*C*t^ (ΔΔ*C*t is the difference between the Δ*C*t of treated samples and that of control samples).

### 4.3. Measurement of ATP1A1-AS1 Subcellular Distribution

Nuclear and cytoplasmic RNA were extracted from cultured HK2 cells using a RNA Subcellular Isolation Kit from Active Motif (Atlanta, GA, USA) (Cat. No. 25501). Extracted RNA was then subjected to reverse transcription for cDNA synthesis and RT-qPCR measurement using the same primer pairs as described above. GAPDH was used as a marker of cytosol RNA as previously reported [47,48] to verify the separation of cytosol and nuclear fraction. Relative expression level in nuclear or cytosol was calibrated according to the total RNA amount obtained from each fraction.

### 4.4. Overexpression of ATP1A1-AS1 and FOXA1 in HK2 Cells

Full length cDNA sequence of *ATP1A1-AS1-203*, Intron 2, or *FOXA1* was cloned into a pcDNA3.1(-B) plasmid vector [49] and was verified by DNA sequencing. About 2 μg per well of the overexpression vector was transfected into HK2 cells cultured in a 6-well plate with X-tremeGENE™ HP DNA Transfection Reagent from Roche (Basel, Switzerland) (Cat. No. 6366236001) for 24 or 48 h. The same amount of empty pcDNA3.1(-B) vector was used as control.

### 4.5. Western Blot

Control or treated HK2 cells were washed with ice cold phosphate-buffered saline (PBS) once and solubilized in Radioimmunoprecipitation assay (RIPA) buffer containing 2m M PMSF, 1% protease inhibitor cocktail, and 1mM sodium orthovanadate from Santa Cruz biotechnology (Dallas, TX, USA) (Cat. No. sc-24948). After centrifuged at 14,000× *g* for 15 min, the supernatants were collected and used for Western blot. The primary antibodies used in western blot analyses were: Anti-Na/K ATPase α1 antibody (Developmental Studies Hybridoma Bank at the University of Iowa, Iowa city, IA, USA, Cat. No. α6F), anti-GAPDH antibody (Santa Cruz Biotechnology, Cat. No. sc-25778), anti-phospho-Src (pTyr418) antibody (Sigma-Aldrich (Saint Louis, MO, USA), Cat. No. S1940), and anti-c-Src antibody (Santa Cruz Biotechnology, Cat. No. sc-8056).

### 4.6. Cell Proliferation Assay

For cell proliferation assay, about 20,000 HK2 cells were seeded in each well on a 12-well plate and cultured for 24 h. The cells were then transfected with pcDNA3.1(-B) vector containing *ATP1A1-AS1-203*. Cells transfected with empty pcDNA3.1(-B) vector were used as control. To monitor the cell proliferation, transfected cells with or without ouabain treatment were incubated for 96 h, in an IncuCyte^®^ S3 Live Cell Analysis System (Essen BioScience)—equipped with a microscope camera that automatically taking pictures from 9 different regions of each well every 2 h. Cell confluences were calculated and analyzed using the IncuCtye S3 live cell analysis software provided by the manufacturer.

### 4.7. Statistics

The data are presented as the Mean ± SEM and analyzed using Two-way ANOVA or Student’s *T*-test where appropriate. A *p*-value < 0.05 is considered as significant.

## Abbreviations

lncRNAs	Long non-coding RNAs
SAHA	Suberoylanilide hydroxamic acid
HDAC	Histone deacetylase
CTS	Cardiotonic steroids
